# Demonstrating the Analytical Potential of a Wearable Microneedle-Based Device for Intradermal CO_2_ Detection

**DOI:** 10.1021/acssensors.3c02086

**Published:** 2024-01-04

**Authors:** Águeda Molinero-Fernandez, Qianyu Wang, Xing Xuan, Åsa Konradsson-Geuken, Gastón A. Crespo, María Cuartero

**Affiliations:** † Department of Chemistry, KTH Royal Institute of Technology, Teknikringen 30, SE-114 28 Stockholm, Sweden; ‡ UCAM-SENS, Universidad Católica San Antonio de Murcia, UCAM HiTech, Avda. Andres Hernandez Ros 1, 30107 Murcia, Spain; § Section of Neuropharmacology and Addiction Research, Department of Pharmaceutical Biosciences, Uppsala University, Uppsala 753 10, Sweden

**Keywords:** ion-selective microneedles, CO_2_ sensing, wearable sensor, interstitial fluid, in vivo measurements, blood correlation

## Abstract

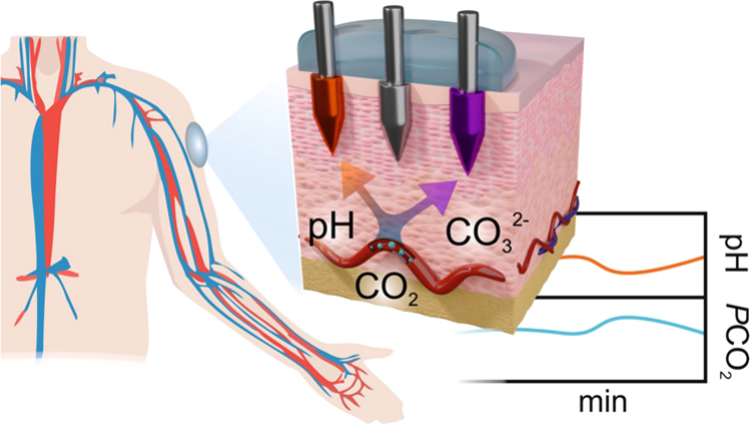

Monitoring of carbon dioxide (CO_2_) body levels is crucial under several clinical conditions (e.g., human intensive care and acid–base disorders). To date, painful and risky arterial blood punctures have been performed to obtain discrete CO_2_ measurements needed in clinical setups. Although noninvasive alternatives have been proposed to assess CO_2_, these are currently limited to benchtop devices, requiring trained personnel, being tedious, and providing punctual information, among other disadvantages. To the best of our knowledge, the literature and market lack a wearable device for real-time, on-body monitoring of CO_2_. Accordingly, we have developed a microneedle (MN)-based sensor array, labeled as CO_2_–MN, comprising a combination of potentiometric pH- and carbonate (CO_3_^2–^)-selective electrodes together with the reference electrode. The CO_2_–MN is built on an epidermal patch that allows it to reach the stratum corneum of the skin, measuring pH and CO_3_^2–^ concentrations directly into the interstitial fluid (ISF). The levels for the pH–CO_3_^2–^ tandem are then used to estimate the *P*CO_2_ in the ISF. Assessing the response of each individual MN, we found adequate response time (*t*_95_ < 5s), sensitivity (50.4 and −24.6 mV dec^–1^ for pH and CO_3_^2–^, respectively), and stability (1.6 mV h^–1^ for pH and 2.1 mV h^–1^ for CO_3_^2–^). We validated the intradermal measurements of CO_2_ at the ex vivo level, using pieces of rat skin, and then, with in vivo assays in anesthetized rats, showing the suitability of the CO_2_–MN wearable device for on-body measurements. A good correlation between ISF and blood CO_2_ concentrations was observed, demonstrating the high potential of the developed MN sensing technology as an alternative to blood-based analysis in the near future. Moreover, these results open new horizons in the noninvasive, real-time monitoring of CO_2_ as well as other clinically relevant gases.

The blood gas analysis is a standard diagnostic tool widely used in intensive care units (ICUs). It provides information about the health status of a patient concerning certain respiratory, circulatory, and metabolic disorders.^1^ Typically, this kind of blood analysis can be performed in any circulatory system (artery, vein, and capillary), providing different types of information, but the most common practice implies arterial blood, resulting in the well-known arterial blood gas (ABG) analysis that grants knowledge related to the respiratory system. The ABG is a very invasive and painful method for adults and indeed unfeasible in neonates, owing to the small blood volume of the newborn.^2^ Despite ABG being the gold standard for the clinical analysis of pH and partial pressures of oxygen and carbon dioxide (*P*O_2_ and *P*CO_2_, respectively), the results are not immediately available because of the blood collection and analysis, which require high levels of expertise from the clinical staff.^3^

About the sampling procedure, once the arterial blood is obtained via an arterial puncture with special needle and syringe, the following main aspects must be considered to avoid any alteration of the blood, and therefore, inaccurate information: (i) unintentional exposure to the air, (ii) in vitro coagulation, (iii) inappropriate selection of the sample container, (iv) optimal temperature for sample storage, (v) the calibration of the analytical instrument (electrochemical sensors composing the ABG instrument) by lab experts, and (vi) the time from the sample collection to its analysis must not exceed the range from 15 to 30 min.^4^ Thus, the entire process is laborious, expensive, slow (single information per day or even lower frequency), with the outcomes being highly dependent on the collection and storage of the sample, as well as calibration condition of the analytical instrument.^5^ Importantly, ICU patients usually require frequent blood gas testing (every second hour in same cases): the most common option for this monitoring is arterial catheterization, which is known to be associated with severe complications, such as arterial/nervous lesions, ischemia, and infections.^5^

From the clinical point of view, the capillary blood (1–2 mm in depth from the skin) and the interstitial fluid (ISF, less than 1 mm in depth from the skin) have been shown to contain significant information about the physiological status of the individual.^6^ Moreover, few systematic studies, mainly focused on glucose, have attempted to discover reliable composition correlations between the bloodstream and those two biological fluids.^6^ In such a context, clinical trials involving pediatric scenarios showed that capillary blood gas (CBG) analysis effectuated at specific conditions may be an alternative for ABG.^7^ Other studies have pointed out that pH and *P*CO_2_ measurements correlate well in venous, arterial, and capillary blood, whereas *P*O_2_ lacks any correlation.^8^ Nonetheless, all of these studies should be carefully considered due to the high risk of change in the gas content of the blood during the entire analysis process.

In a different direction, noninvasive methods for CO_2_ detection have been developed to overcome the above-mentioned issues related to ABG (and analogous techniques applied to other blood samples), such as the end-tidal approach, a colorimetric CO_2_ detector for exhalated air and transcutaneous CO_2_ (tcCO_2_) measurements, though all of these exhibit important applicability limitations yet.^5^ Regarding tcCO_2_, the amount of CO_2_ diffusing through the skin is detected. Briefly, the sensor, which is a pH electrode in contact with a solution delimited by a thin CO_2_ permeable membrane (Stow–Severinghaus electrode), is placed onto the skin surface. The CO_2_ that diffuses across the skin (after warming) passes through the membrane and dissolves in the solution, causing a pH change that is registered by the pH electrode. The tcCO_2_ technique is painless, without the necessity of blood extraction, and has also presented a good correlation with ABG values.^5^ However, the precision of the measurements has been questioned from data obtained in certain setups, such as emergency rooms and ICUs. The need for warming the skin at above 42 °C results in both skin burns (especially in neonates) and deterioration of the sensor itself.^9^ The need for continuous recalibration (every few hours in some cases), given the low stability of the sensor over time, is another inconvenience.^9^

Considering sensing devices capable of working under the stratum corneum of the skin, microneedle (MN) technology has been positioned as an elegant approach for measuring in the ISF. Even though significant progress has been recently demonstrated in MN-based electrochemical sensors (by our group and others^10−15^), its implementation for gas monitoring remains underexplored. An example found in the literature is based indeed on an optical MN for intradermal O_2_ monitoring in pig skin.^16^ To the best of our knowledge, neither electrochemical nor optical MNs have been proposed for the assessment of *P*CO_2_ so far. Herein, we present the development of a new MN-based sensing concept for the minimally invasive transdermal detection of CO_2_. It is a potentiometric CO_2_–MN system that consists of three all-solid-state ion-selective MNs: a carbonate-selective MN (CO_3_^2–^–MN), a pH-selective MN (pH–MN), and a reference MN (RE–MN). When implemented in an epidermal patch, the CO_2_–MN system penetrates the stratum corneum of the skin, measuring directly into the ISF and without the need of generating CO_2_ diffusion, in contrast to the tcCO_2_ method. Having simultaneous measurements of pH and CO_3_^2–^ in the ISF, concentrations of bicarbonate and *P*CO_2_ can be estimated. Accordingly, this work demonstrates the accurate detection of *P*CO_2_ in ISF with a potentiometric MN–CO_2_ system, including the in vivo feasibility demonstration with anesthetized rats. Also, the correlation between blood and ISF CO_2_ measurements is assessed to investigate the potential to substitute ABG in clinical settings in the near future.

## Experimental Section

### Reagents and Materials

Hydrogen ionophore I (tridodecylamine) of selectophore grade, carbonate ionophore VII (*N*,*N*-dioctyl-3α,12α-bis(4-trifluoroacetylbenzoyloxy)-5β-cholan-24-amide) of selectophore grade, poly(vinyl chloride) (PVC), polyurethane (PU) (Reference 81367, selectophore grade), bis(2-ethylhexyl)sebacate (DOS, >97%), sodium tetrakis[3,5-bis(trifluoromethyl)phenyl]borate (NaTFPB), tridodecylmethylammonium chloride (TDMACl), bis(2-ethylhexyl) adipate (DEHA), and tetrahydrofuran (THF, >99.9%) were purchased from Sigma-Aldrich (Sweden). Carbon/graphite ink (C2030519P4) and silver/silver chloride (Ag/AgCl) 50/50 paste (C2131007D3) were obtained from Sunchemical.^17,18^ Silicon rubber (Ecoflex 00–50 platinum cure) and stainless-steel microneedles (MN) (Dermaroller local supplier, Sweden) were employed for the fabrication of the MN patch. Other chemicals and materials are provided in the Supporting Information.

### Fabrication of the MN Patch for CO_2_ Detection

The CO_2_–MN patch consisted of a flexible substrate made of a 1 mm deep silicone rubber in which three solid stainless-steel MNs (1500 μm in length and 150 μm in diameter) were placed and then conveniently modified: the upper MN part was used to make the electrical connections to the reader (potentiometer), while the tip part (500 μm in length after being fixed in the substrate) was conveniently modified to provide the sensing capabilities. Two of the MNs were modified to create two working electrodes (WE–MNs) consisting of ion-selective electrodes with plasticized polymeric membranes that are selective for pH or CO_3_^2–^. Then, the third MN was modified to provide a common reference electrode (RE–MN) for the potentiometric measurements. Both WEs were of the all-solid-state format, prepared with three layers deposited onto the stainless-steel MN structure. From the inside to outside part: (i) a carbon ink layer to improve the conductivity of the MN and the adherence of the next layer to the MN; (ii) lipophilic (functionalized) multiwalled carbon nanotubes (f-MWCNTs)^14^ as the ion-to-electron transducer; and (iii) the corresponding ion-selective membrane (ISM) to provide selectivity and the potential response. The RE-MN was also of the all-solid-state format and consisted of an Ag/AgCl layer covered by a reference membrane (RM) and an external PU layer, as reported elsewhere.^14^

**Figure 1:**
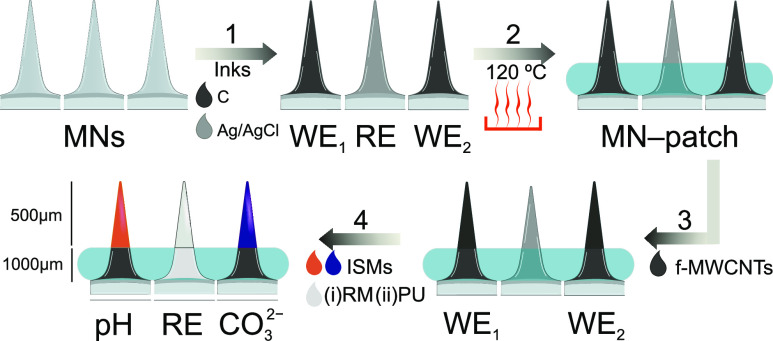
Scheme of the procedure for the preparation of the MN-CO_2_ patch: (1) stainless-steel solid MNs are modified with commercial inks to obtain two WE-MNs and one RE-MN; (2) the MNs are cured in an oven and assembled into the silicon substrate; (3) deposition of f-MWCNTs on top of the carbon ink in the WE-MNs; (4) deposition of the corresponding ion-selective membrane (ISM) in the WE-MNs, and the reference membrane (RM) in the RE-MN, and deposition of an extrapolyurethane (PU) layer in the RE-MN.

Figure 1 depicts a scheme of all of the steps accomplished toward the preparation of the MN-CO_2_ wearable patch. First, the tips of the two MNs to be used as the WEs were covered by a film of carbon by dip-coating them into a commercial ink. A similar procedure was followed to prepare the RE-MN but covering the MN with Ag/AgCl. The three MNs were cured in an oven at 120 °C for 10 min. Then, the three (coated) MNs were inserted into the substrate, and the upper part of each MN was glued to the rubber with Loctite Super Glue (Henkel Norden AB). After drying the glue for 20 min at room temperature, the remaining functionalization steps were accomplished in the tip part of each (coated) MN.

For the WEs, 10 subsequent layers with a volume of 2 μL of an f-MWCNTs dispersion in THF (1 mg mL^–1^) were drop-cast onto the carbon MN.^10,14^ Drying steps of 4 min were performed between layers. The excess of f-MWCNTs after each drop deposition was carefully wiped off using a micropipette. Then, 3 layers of a volume of 1 μL of the corresponding ISM (the compositions of the cocktails are provided in the Supporting Information) were drop-cast. Drying steps of 20 min were performed after the addition of the first and second layers and 4 h after the deposition of the final layer to ensure the appropriate drying (i.e., THF evaporation) of the membrane. Finally, both WE–MNs were conditioned overnight. The pH-MN was conditioned in 1 mM HCl and the CO_3_^2–^–MN was conditioned in 1 mM NaHCO_3_. For the RE, 3 layers of a volume of 1 μL of the reference membrane (RM) cocktail (see the composition in the Supporting Information), were drop-cast onto the Ag/AgCl MN. Drying steps of 20 min after the addition of the first and second layers and 4 h after the incorporation of the final layer were performed. Then, after overnight conditioning in a 3 M KCl solution, the RE was dried at room temperature for 1 h. An extra layer of PU (2 μL, 20 mg mL^–1^ in THF) was drop-cast, and the MN was conditioned in 3 M KCl for 12 h.

### Ex Vivo Measurements in Pieces of Rat Skin

For the ex vivo experiments, 6 pieces of rat skin (approximately 5 cm × 5 cm, Biobreeder rats) were overnight conditioned in solutions of artificial interstitial fluid (AISF) containing different, known concentrations of CO_2_ (0.2–3 mM). After that, the corresponding piece of skin was placed into a plastic support and covered with a piece of parafilm with the same dimensions as the MN patch to allow for MN-CO_2_ patch insertion while isolating the rest of the skin (Figure S1). The skin pieces were obtained from euthanized rats and were donated by the Karolinska Experimental Research and Imaging Centre (Karolinska Institutet, Stockholm, Sweden).

### In Vivo Measurements in Anesthetized Rats

Five anesthetized rats were employed for in vivo assays (see the Supporting Information). Prior to in vivo measurements, each awake rat was placed into an anesthesia induction chamber for initial anesthesia (isoflurane/air mixture, 2–4 L/min, 4% of isoflurane). After that, each rat was placed in an anesthesia mask, and the isoflurane level was adjusted according to 2–4 L/min isoflurane/air 2–2.5% v/v during the experiments. Any change in the anesthesia composition was recorded. A heating pad was placed under the animal during the whole experiment, and artificial tears (Viscotears, Bausch Lomb Nordic, Stockholm, Sweden) were applied to the eyes of the rat to lubricate the ocular surface when tear production is reduced due to the anesthesia. A small part of the back of the rat was shaved and sterilized with ethanol for MN insertion.

In parallel to the anesthesia and rat preparation procedure, the MN-CO_2_ patch was calibrated by a 3-point procedure in AISF background. Notably, the solutions’ pH and CO_2_ levels were checked with commercial sensors to be exactly incorporated in the calibration graphs. The pH meter (Metrohm, Sweden) and Severinghaus probe (ThermoFisher) were used for this purpose. Then, the skin of the rat was carefully pierced with the CO_2_–MN patch and the data was recorded for approximately 2 min. After the on-body acquisition, the rat was physically euthanized, and a blood sample was collected from the incision in the neck and analyzed with a portable blood gas analyzer (i-Stat 1). These experiments were approved by and conducted in accordance with the Uppsala Committee on Ethics of Animals (Dnr 5.8.18- 18873/2018, DOUU-2020–025). More details on the procedures are provided in the Supporting Information.

## Results and Discussion

### Principle for CO_2_ Detection with the CO_2_–MN Patch

**Figure 2:**
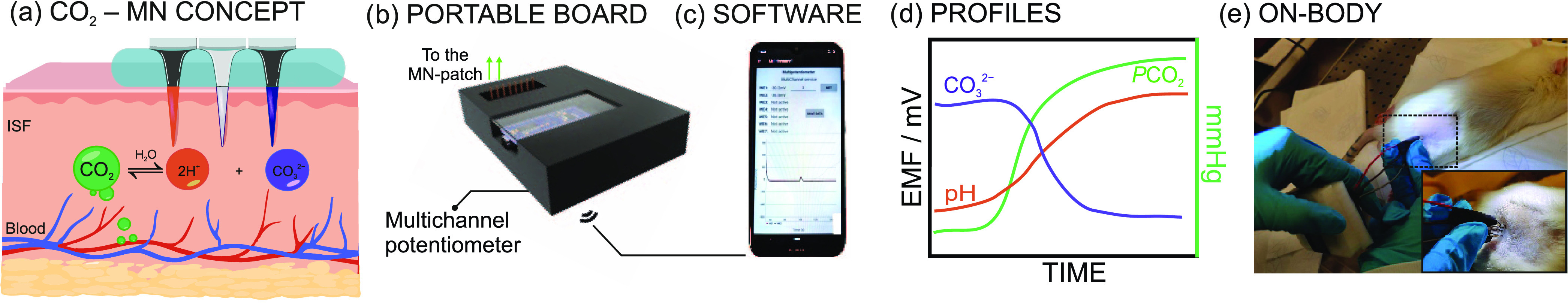
(a) Conceptual scheme of the CO_2_–MN device and its working principle. (b) Image of the portable multichannel board embedded in a 3D-printed casing. (c) Custom-made software employed for the on-body measurements. (d) Dynamic profiles for pH and CO_3_^2–^ measured with the patch, and the *P*CO_2_ profile calculated based on such measurements. (e) Image of on-body measurements in anesthetized rats.

The CO_2_–MN patch consisted of two WE–MNs, labeled as a pH–MN and CO_3_^2–^–MN, and a shared RE–MN. The three MN–based electrodes were embedded into a silicone rubber substrate with a circular shape (Ø = 1 mm) forming the final CO_2_–MN patch (Figure 2a). The MNs were connected to a miniaturized custom-made multipotentiometer board for signal acquisition and processing, being in turn wirelessly connected to the user interface in a mobile phone through Bluetooth (Figure 2b). The portable board was placed inside a 3D-printed casing for protection and better handling once connected to the MNs. The user interface was a custom-made mobile app to display, analyze, and store the real-time potentiometric signals obtained by the CO_2_–MN patch (Figure 2c). Figure S2a,b show real pictures of the CO_2_–MN patch and the entire device (i.e., MNs attached to the electronic board) when on-body measurements were performed in anesthetized rats.

Figure 2d exemplifies the dynamic measurements of pH and CO_3_^2–^ with the MNs together with the calculated *P*CO_2_ profile. Importantly, the CO_2_ (and *P*CO_2_) detection relies on the potentiometric signals independently provided by the pH–MN and the CO_3_^2–^–MN sensors, as the following explained. The potentiometric signal of each MN is proportional to the H^+^ (pH) and CO_3_^2–^ concentrations in the sample (i.e., considering concentration equal to activity in the AISF or ISF background). Thereafter, CO_2_ concentration can be estimated according to eq 1
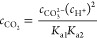
1where *c*_co_3_^2–^_ and *c*_H^+^_ are the CO_3_^2–^ and hydrogen-ion concentrations, respectively, and *K*_a1_ and *K*_a2_ are the first and second dissociation constants for carbonic acid. Then, it is known that the concentration of a gas dissolved in a liquid is proportional to the partial pressure of the gas under equilibrium conditions. Thus, *P*CO_2_ can be obtained from the concentration of dissolved CO_2_ using the following equation
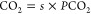
2where *s* is the solubility constant for CO_2_ in the medium.^19^

Notably, the use of two potentiometric probes to indirectly estimate the CO_2_ levels was explored in various applications. For example, submergible probes based on CO_3_^2–^ and pH sensors were settled to monitor species of the carbon cycle in seawater.^20,21^ Also, Kraig et al. demonstrated CO_3_^2–^ and pH inner-filling microelectrodes for the detection of CO_2_ in the extracellular fluid of rat hippocampal slices,^22^ and intracellular CO_2_ measurements in skeletal muscle cells in anesthetized rats.^23^ However, to the best of our knowledge, this sensing strategy has not yet been proposed for intradermal measurements with MNs in view of replacing ABG tests, as established in this work.

In view of executing proper on-body measurements in rats (Figure 2e), the MNs in the patch must reach the ISF while maintaining the integrity of the sensing elements and following a painless procedure for the individual. Effectively, the mechanical characteristics of the patch and the MNs (e.g., length of the MN, number of MNs, diameter at the MN base, diameter at the tip, and tip angle of the MN) influence both the piercing capabilities and the patient’s comfort.^24^ Accordingly, SEM images were acquired to investigate the suitability of the developed CO_2_–MN patch for on-body measurements. The images are presented in Figure S3 in the Supporting Information. Both types of MNs, WE–MNs (CO_3_^2–^–MNs is depicted as an example) and the RE–MN, presented a length that ensures to reach the dermis (500 μm), a base diameter of <300 μm, tip diameter of <50 μm, and tip angle of <45°. These features agreed with already established thresholds for proper insertion into the skin while ensuring the user well-being.^24^

### In Vitro Characterization of the Analytical Performance of the MNs for pH and CO_3_^2–^ Detection

First, the analytical performances of the MNs for the detection of pH and CO_3_^2–^ were studied by means of an in vitro configuration. The potentiometric responses at varying pH and CO_3_^2–^ concentrations were recorded for the corresponding WE-MN fixed in the patch and against a commercial double-junction Ag/AgCl reference electrode. Buffer solutions in the pH range from 5.0 to 8.5 were used to calibrate the pH-MN. For CO_3_^2–^, solutions with increasing concentrations of NaHCO_3_ were prepared at pH = 7.4 (HEPES buffer background), in agreement with the physiological pH. Notably, the pH of the solutions was constantly monitored with the pH meter, and the final CO_3_^2–^ concentrations were calculated according to the acidic constants established for H_2_CO_3_ at 25 °C in water (p*K*_a1_ 6.35 and p*K*_a2_ 10.33).^25^

Close-to-Nernstian slopes were obtained for both WE-MNs: 53.9 ± 1.0 mV pH^–1^ for pH (*n* = 3), and −26.6 ± 0.5 mV dec^–1^ for CO_3_^2–^ (*n* = 3). The linear range of response (LRR) was observed from 5 to 8.5 for pH, which completely covers the expected values in ISF. Even though the arterial blood pH stays in the range of 7.35–7.45 under normal conditions, the pH of ISF seems to be more unstable and may vary in connection to some diseases. For example, acidic pHs (as low as 6.5) have been observed in diabetes and cancer clinical conditions, whereas alkaline pHs up to 8.5 have been reported for certain wounds.^26^ The LRR for CO_3_^2–^ was obtained from 0.37 μM to 3 mM, which includes the levels expected in arterial and venous blood as well as ISF (Table S1). For both WEMNs, fast responses (*t*_95_ < 5 s), good repeatability (RSD ≤ 2% for the slope and ≤1% for the intercept, 3 calibrations using the same MNs) and adequate between-electrodes reproducibility (RSD ≤ 4% for the slope and ≤25% for the intercept, 3 analogous MNs) were obtained (Table S2).

**Figure 3:**
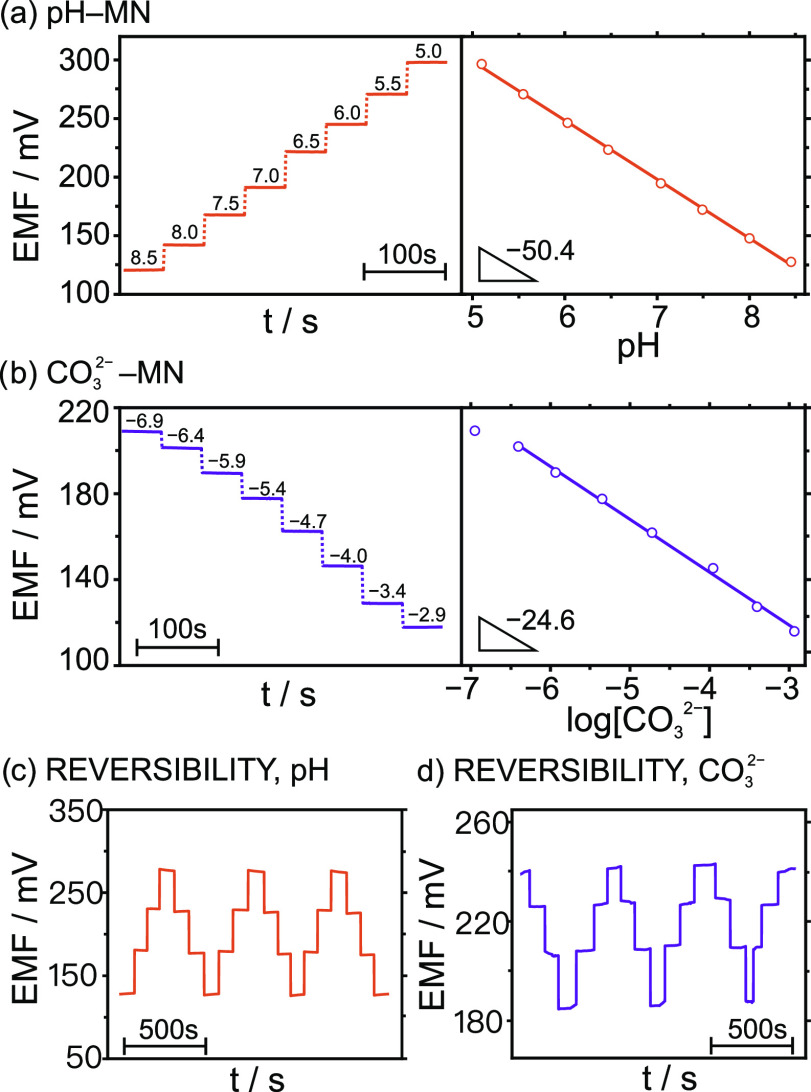
(a) Left: dynamic response of the pH–MN against the RE-MN at decreasing pH in the AISF background. Right: the corresponding calibration graph. (b) Left: dynamic response of CO_3_^2–^–MN against the RE–MN at increasing CO_3_^2–^ concentrations in the AISF background. Right: The corresponding calibration graph. (c, d) Reversibility study of the pH– and CO_3_^2–^–MN responses upon decreasing and increasing pH (8.0, 7.0, 6.0, and 5.0) and increasing and decreasing CO_3_^2–^ levels (10^–6.0^, 10^–5.0^, 10^–4.0^, and 10^–3.0^ M) in the sample against the RE-MN.

Next, we proceeded to substitute the commercial Ag/AgCl reference electrode by REMN, and the analytical parameters were re-evaluated. Advantageously, no significant changes were observed concerning the response of three analogous patches (Table S3) beyond a change in the displayed potential range, which is inherent to the change in the RE nature and mainly affected the intercept values for the calibration graphs. Moreover, the stability of the response was evaluated in artificial solutions at fixed pH and CO_3_^2–^ concentrations (Figure S4), showing acceptable medium-term drifts over 7 h of 0.9 ± 1.1 mV h^–1^ in phosphate buffer at pH 7.4 and −0.8 ± 2.3 mV h^–1^ in 20 mM NaHCO_3_, for pH and CO_3_^2–^ MNs, respectively. A selectivity study was performed by considering the major ions found in ISF (Table S4) that may interfere in the potentiometric response. Other components (such as urea, glucose, amino acids, lactate, and ascorbic acid) are not expected to affect the measurements. Selectivity coefficients were estimated by using the separate solution method (SSM), in which individual calibration graphs are obtained for both the potential interference and the primary analyte (pH or CO_3_^2–^ in our case). Notably, as previously established, these values have to be considered as “apparent”, since the interferent ions did not present Nernstian slopes and the SSM is based in such an assumption.^27^ A comparison of the estimated logarithmic selectivity coefficients with those theoretically required to perform potentiometric measurements in ISF without any expected interference revealed the suitability of the CO_2_–MN patch for such measurements (Table S5). Moreover, the selectivity coefficients agreed well with those reported for other MNs for pH, as well as ISEs based on the same ionophores as those used herein. Additionally, calibration curves were carried out in AISF media, the composition of which is provided in the Supporting Information, for a complete matrix interference study. Figure 3a,b presents the results. No significant differences in the slopes were observed compared to the previous results in buffered background (−26.4 ± 0.8 mV dec^–1^ and −51.2 ± 0.7 mV pH^–1^), and similar LRRs were obtained (5.0–8.5 for pH, and 10^–6.0^–10^–2.6^ M for CO_3_^–2^), covering the expected concentrations in ISF (Table S2). These results suggested that the developed MNs can be used for the further analysis of real ISF.

The ability of the MNs to accurately detect time-based fluctuations in the pH and CO_3_^–2^ was assessed. This is important to ensure continuous monitoring of the patient’s state with the CO_2_–MN device. A reversibility test was performed by subsequently decreasing and increasing the pH in the range from 8 to 5, and increasing and decreasing the CO_3_^–2^ concentration from 10^–6.0^ to 10^–3.0^ M. The registered dynamic potentials are depicted in Figure 3c,d, respectively. In both cases, the MNs displayed a fully reversible signal with a variation of the slope of about 2.4% (%RSD) for pH, and 1.6% for CO_3_^–2^, and variations for the intercept of 2.5% for pH and 2.7% for CO_3_^–2^. Notably, this study was conducted considering very wide concentration changes, which are not indeed expected to occur in real biological systems but served to fully understand the benefits and limitations of the developed CO_2_–MN patch.

The stabilities of the MNs’ responses were tested in AISF media over 7 h (see Figure S4). Higher drifts were observed in AISF than in buffer (2.1 ± 2.3 mV h^–1^ for CO_3_^2–^ and 1.6 ± 0.4 mV h^–1^ for pH). Regarding how these drifts could affect the accuracy of further on-body measurements, while a threshold of 10% in the CO_2_ concentration is clinically accepted, which will be covered with the CO_2_–MN patch over the first ca. 30 min, the drift may affect the system for relatively long assays (>1 h). Errors >20% may happen and therefore, recalibration every hour or the patch substitution could be a solution to avoid any inaccuracy issue, considering the case that such measurements are clinically relevant and therefore needed. Overall, the CO_2_–MN patch presented excellent analytical features that are promising for reliable in vivo intradermal analysis of CO_2_.

### In Vitro Investigation of the Determination of CO_2_ from the pH–CO_3_^2–^ Measurements Obtained with the CO_2_–MN Patch

**Figure 4:**
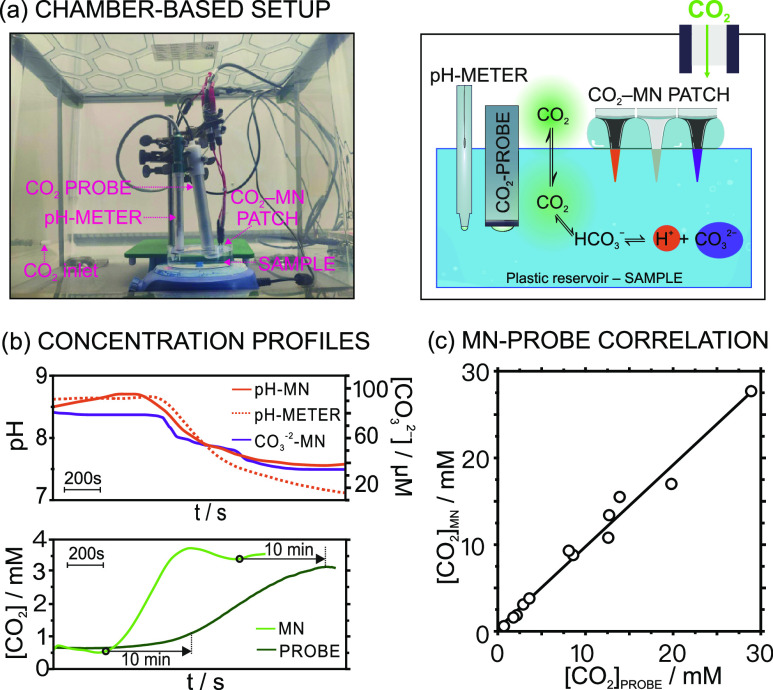
(a) Photo and scheme of the experimental setup for CO_2_ monitoring in sample solutions placed inside the isolation chamber. (b) pH, CO_3_^2–^, and CO_2_ dynamic profiles observed when the CO_2_ concentration was increased inside the chamber. The CO_2_–MN patch, pH meter, and Severinghaus probe were used to monitor the process. Dashed lines with arrows indicate the lag times for CO_2_ measurements for the Severinghaus probe. (c) Correlation between the CO_2_ values measured in artificial samples with the CO_2_ MN patch and the Severinghaus probe (*n* = 13).

To demonstrate the possibility of monitoring real-time changes in CO_2_ in both buffer and AISF samples, the CO_2_–MN patch was tested in the setup presented in Figure 4a. In essence, the patch was immersed in a sample solution (25 mM NaHCO_3_ or AISF), which is contained in a plastic reservoir, together with the commercial micro pH meter and the Severinghaus CO_2_ probe. These two latter electrodes served to validate the profile of CO_2_ levels calculated from the pH–CO_3_^2–^ tandem measurements. The CO_2_ probe was positioned with a ca. 20° angle from the vertical to avoid the trapping of air bubbles at the tip of the electrode (as suggested by the supplier). The setup was in turn placed inside a plastic (isolation) chamber with a CO_2_ gas inlet and outlet, and then the recording of all of the sensors was started. Notably, all of the sensors were calibrated out of the chamber before and after the experiment, in case of need for possible drift correction due to the long duration of the experiments. Once all of the sensors presented a stable signal in the sample solution (ca. after 100 s of having started the measurements), this was exposed to increasing CO_2_ concentrations by introducing the gas into the chamber. The sample was allowed to re-equilibrate with the CO_2_ partial pressure achieved in the atmosphere, while concentration changes were monitored by all of the sensors.

The dynamic CO_2_ concentration in the solution was calculated according to eq 1, from the dynamic pH and the CO_3_^2–^ concentration provided by the MN patch. The top plot in Figure 4b shows the concentration profiles for pH and CO_3_^2–^ directly measured with the CO_2_–MN patch, together with the estimated profile for CO_2_, in one of the experiments performed in 25 mM NaHCO_3_ solution (as an example). It was evidenced how the introduction of CO_2_ into the chamber translated into a gradually higher CO_2_ concentration in the sample solution, with decreasing pH and CO_3_^2–^ levels. Indeed, the pH profile agreed rather well with that displayed by the pH meter.

A comparison between the CO_2_ profiles provided by the MN patch and the Severinghaus probe (the bottom plot in Figure 4b) revealed interesting hints. Despite both profiles being qualitatively similar, a slower response time of ca. 10 min was shown by the Severinghaus probe with respect to the MNs (illustrated with dashed arrows in Figure 4b). This is indeed expected since measurements based on the Severinghaus concept consider pH changes in the inner compartment of the probe, to which CO_2_ is transported from the sample solution across a selective outer diffusion-limiting membrane.^5^ Accordingly, the Severinghaus CO_2_ probe may be utilized as a reference method to validate the CO_2_ results provided by the MN patch only before and after the amount of CO_2_ was increased in the sample solution, when both devices displayed constant and comparable responses considering a lag time of ca. 10 min.

Table S6 presents the CO_2_ levels obtained by the CO_2_–MN sensor and the commercial Severinghaus CO_2_ probe after different increases in CO_2_ concentration in both 25 mM NaHCO_3_ solution and AISF. Notably, profiles and trends different from those just described for buffer samples (i.e., profiles in Figure 4b) were observed in AISF. In essence, phosphate species are the main factors responsible for the buffer capacity in the AISF (pH = 7.4) and thus, the addition of the CO_2_ in the chamber induced an increase of the HCO_3_^–^ concentration without drastically affecting the (buffered) pH. Accordingly, there was an increase in the HCO_3_^–^/H^+^ molar ratio that produced in turn an increase in the CO_3_^2–^ concentration (Figure S5) and hence, the CO_2_.^28^

Overall, the differences between the outcomes from the CO_2_–MN patch and the reference method were <15% (threshold established for the validation of an analytical methodology),^29,30^ confirming the acceptable accuracy of the measurements. Larger differences were observed only in two samples, which indeed presented relatively high CO_2_ concentrations (samples #8 and #15). Figure 4c shows the correlation between the CO_2_ concentrations provided by the CO_2_–MN patch and the CO_2_ probe (sample size of *n* = 13, without considering samples #8 and #15). A line with a *y*-intercept of 1.3 mM (rather close to zero), a slope of 0.8 (close to one), and a Pearson coefficient of 0.98 (*p* < 0.05) were found, revealing the existence of a positive correlation. Despite these acceptable results, some aspects should be considered with regard to the evaluation/discussion of the CO_2_-MN patch accuracy. On the one hand, at low CO_2_ levels (0.1–0.5 mM), it has been reported that the precision of the Severinghaus probe is affected by a deviation from the (ideal) Nernstian response.^31^ Effectively, there is a sensitivity worsening due to the deteriorating buffer capacity of the HCO_3_^–^/CO_2_ couple, as already reported.^31^ On the other hand, at high CO_2_ levels, the corresponding CO_3_^2–^ concentration may be close to the lower LOD (i.e., 1.2 μM) of the CO_3_^2–^–MN, and hence, higher errors are likely to be obtained. Undoubtedly, the results demonstrated the feasibility and good reliability of measuring CO_2_ with the CO_2_–MN patch within the expected clinical range.

### Ex Vivo Analytical Characterization of the CO_2_–MN Patch

Two aspects were evaluated within the ex vivo assays: (i) the resilience of the MN sensors’ response to skin insertion, and (ii) the accuracy of transdermal measurements of CO_2_. First, the resiliency of the MN sensors when fixed in the patch was evaluated by comparing the corresponding calibration graphs before and after one and three insertions into pieces of rat skin. Although no significant changes were observed for the slopes (RSDs < 5%), a slight gradual shift of the intercept to a lower potential was displayed, being this change more significant after three insertions and in the case of the CO_3_^2–^–MN than for the pH–MN (Figure S6, RSDs of 8.7 and 5.9% for CO_3_^2–^ and pH). Accordingly, a recalibration of the MN sensors is advisable if the patch is desired to be used for more than one insertion.

In the second part of the study, the CO_2_ contents in six pieces of rat skin that were 24-h-conditioned in solutions with different carbonate concentrations (200, 90, 50, 10 μM) and pHs (8.0, 7.7, 7.4, and 7.0) were transdermally detected with the MN patch. The setup described in the Experimental Section and Figure S1 was used in the experiments. The CO_2_–MN patch was calibrated and then inserted into a skin sample, covered with a parafilm layer to shield the setup from the atmosphere, thus minimizing the diffusion of CO_2_ across the skin. The transdermal CO_3_^2–^ concentration and pH were calculated from the potential readouts of the corresponding MN and calibration graphs. Finally, the CO_2_ content was estimated from these two measurements, as explained in the previous section. In addition, the ISF inside each of the six skins was collected via a homemade extraction system composed of a hollow MN hub connected to a syringe pump (Figure S7) and analyzed with the reference techniques. Owing to the low volume of extracted ISF, the Severinghaus probe was not suitable for the validation of these measurements. Also, in most of the cases, the collected ISF volume was not enough for employing the portable blood gas analyzer device (Abbott i-Stat 1, a volume ≥95 μL is needed). Thus, the ISF samples were analyzed with a combination of an ultra-micro pH meter, and a CO_3_^2–^–MN, requiring ca. 5 μL of sample.

**Figure 5:**
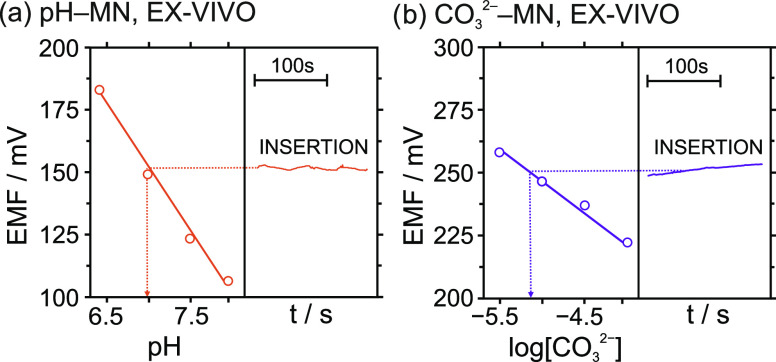
Calibration graphs and dynamic potentials observed for the (a) pH– and (b) CO_3_^2–^–MNs in the CO_2_-MN patch performing the ex vivo experiments with skin #5.

Figure 5 presents an example of the calibrations obtained for the pH– and CO_3_^2–^–MNs together with the dynamic potential profiles observed during the patch insertion in skin #5. As observed, the transdermal readouts presented by both MNs were rather constant at the experimental time scale of the skin insertion (ca. 2 min), and thus, the averaged potentials were extrapolated to the corresponding calibration graph for the calculation of pH, CO_3_^2–^ and CO_2_ levels. The pH and the CO_3_^2–^ and CO_2_ contents provided by the CO_2_–MN patch were compared with those obtained by the reference methods in the analysis of the ISF samples collected from each skin. Table 1 displays all of the data as well as the differences (in %) between the MNs and the corresponding reference technique.

**Table 1:** Results in the Ex Vivo Analysis Utilizing Pieces of Rat Skin^a^

	conditioning solution		CO_2_–MN patch			reference method			differences (%)		
skin sample	pH	CO_3_^2–^ (μM)	pH^b^	CO_3_^2–^ (μM)^c^	CO_2_ (mM)^d^	pH	CO_3_^2–^ (μM)	CO_2_ (mM)	pH	CO_3_^2–^	CO_2_
1	7.96	200	8.05	247.3	0.5	8.0	ND	<0.2^e^		0.6	
2	7.72	90	7.72	59.3	0.8	7.73	72.1	0.8	0.1	17.8	0
3	7.71	90	7.69	80.9	1.2	7.78	90.9	1.0	1.2	11.0	20.0
4	7.35	50	7.43	49.6	2.4	7.51	58.1	2.3	1.1	14.6	4.3
5	7.04	10	6.94	8.8	4.1	7.03	10.2	3.5	1.2	13.7	17.1
6	6.95	10	7.03	2.1	6.2	ND	ND	ND			

a ND = non detecting content, because the collected volume of the ISF was not enough to perform the measurements.

b Ultra-micro pH-meter.

c CO_3_^2–^–MN.

d Calculated from pH and CO_3_^2–^ measurements.

e i-Stat Abbott (*P*CO_2_ < 5 mmHg).

Acceptable results were obtained for samples #2–#5, with averaged percentages for the differences of <20%: 0.9 ± 0.5 for pH, 14.2 ± 2.8 for CO_3_^2–^ and 10.3 ± 9.7 for CO_2_. Additionally for this set of samples, a paired sample *t* test was carried out. No statistical differences at the 95% of confidence interval between the intradermal and collected ISF values were found for pH (*p* = 0.19), and CO_2_ (*p* = 0.18). Then, the ISF sample collected from skin #1 was the only one analyzed with the ABG device, providing a value of *P*CO_2_ < 5 mmHg that approximately corresponds to a CO_2_ concentration <0.15 mM. The precision for such a value was not enough to be quantitatively compared with the results provided by the CO_2_–MN patch (0.5 mM) since it was below the limit of detection of the ABG device. Regarding skin #6, an extremely low volume of ISF (<5 μL) was extracted, hence precluding its analysis. Overall, the accuracy of the intradermal measurements using the CO_2_–MN results in the *ex vivo* approach showed suitability for further animal-based tests, in particular, with anesthetized rats.

### In Vivo CO_2_ Measurements in Anesthetized Rats

**Figure 6:**
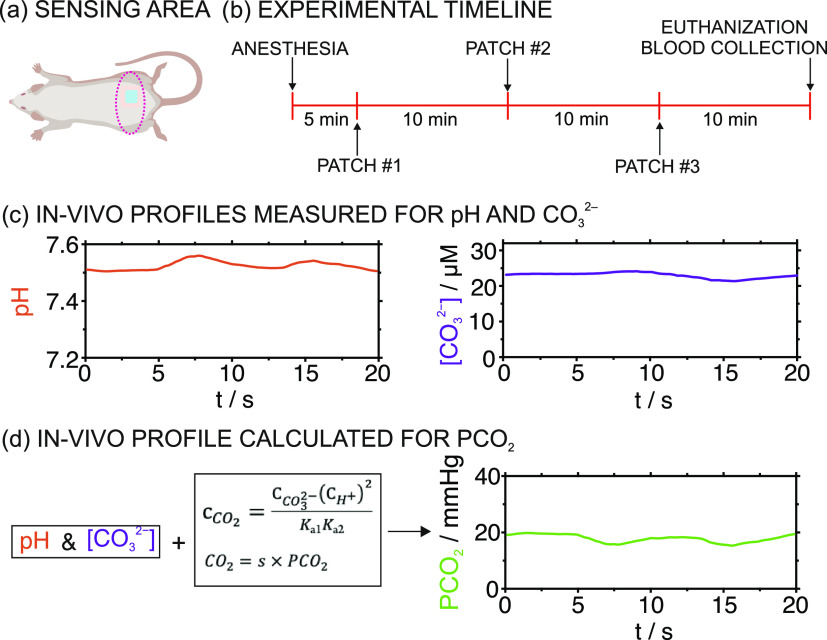
In vivo measurements in rats with the CO_2_–MN patch. (a) Sensing zone. (b) Experimental timeline. (c) Dynamic profiles for pH and CO_3_^2–^ obtained in rat no. 2 at 15 min from the anesthesia. (d) The *P*CO_2_ profile calculated from pH, CO_3_^2–^, eqs 1 and 2.

The developed CO_2_–MN patch was used for in vivo monitoring of CO_2_ levels in the ISF of five anesthetized rats. Details about the experiments, protocols, and ethical permit are provided in the Experimental Section and Supporting Information. In essence, the CO_2_–MN patch was connected to the portable multipotentiometric board inside the casing and calibrated. Once the rat was successfully anesthetized and positioned in the assay platform (ca. 5 min after the initiation of the anesthesia), the MNs were manually inserted into the lower part of the back of the rat (Figure 6a), which was previously shaved (Figures 2e and S2b), and transdermal measurements for ca. 2 min were allowed. The entire assay lasted ca. 40 min for each rat, with discrete measurements performed at ca. every 10 min with different patches. The experimental timeline is illustrated in Figure 6b. After the on-body measurements, the rat was physically euthanized, and samples containing a mixture of arterial-venous blood were collected and analyzed with the blood gas analyzer device immediately after collection and after 3 h (to discard any change in *P*CO_2_ due to atmospheric equilibration, Supporting Information and Table S7).

Figure 6c depicts the dynamic pH and CO_3_^2–^ concentration profiles obtained for rat 1, as an example. The combination of these two by considering eq 1 provides the dynamic CO_2_ concentration. Additionally using eq 2 and the appropriate solubility product (Supporting Information*)*, the *P*CO_2_ profile can be estimated (Figure 6d). Then, to investigate if these measurements correlate with the traditional blood gas test, discrete data points for pH, bicarbonate (HCO_3_^–^), and *P*CO_2_ were calculated by averaging 20 s of the entire responses provided by the pH– and CO_3_^2–^–MNs (i.e., portions of stable signal occurring after the initial response time of the MNs, ca. 30 s after the measurement initiation, were selected). Then, in addition to *P*CO_2_ and pH values, the blood gas analyzer displays the HCO_3_^–^ levels; therefore, these were also calculated from the MNs for comparison purposes. While *P*CO_2_ is mainly balanced by the respiratory system and gives information related to patient’s ventilation, HCO_3_^–^ is mainly regulated by the kidney, and indicates the presence of metabolic disorders. Altogether may provide valuable clinical insight about acid–base disturbances.^32^ The reader is kindly referred to the Supporting Information about HCO_3_^–^calculations performed herein from the experimental data.

Table 2 shows the results for the in vivo measurements performed with the six rats together with those observed in the blood gas test. In ISF, excluding the data in rat no. 4, pH values ranged from 7.2 to 7.8, HCO_3_^–^ from 11 to 25 mM, and *P*CO_2_ from 10 to 74 mmHg. In blood, pH values ranged from 7.3 to 7.7, HCO_3_^–^ from 14.0 to 30 mM, and *P*CO_2_ from 19 to 54 mmHg. In principle, ISF and blood ranges were rather coincidental and within the expected clinical levels (Table S7). Therefore, further correlation and statistical analyses were performed, except for the data from rat no. 4 that presented a high difference between ISF-blood results.

**Table 2:** Results in the In Vivo Analysis in Five Anesthetized Rats^a^

		ISF					blood		
rat no.	patch number/time (min)^b^	pH	CO_3_^2–^ (μM)	HCO_3_^–^ (mM)	CO_2_ (mM)	*P*CO_2_ (mmHg)	pH	HCO_3_^–^ (mM)	*P*CO_2_ (mmHg)
1	1/15	7.52 ± 0.02	23.0 ± 0.7	10.8 ± 0.5	0.5 ± 0.04	17.3 ± 1.3	7.7	23.1	18.8
	2/25	7.61 ± 0.07	45.1 ± 0.9	18.3 ± 0.1	0.6 ± 0.1	18.8 ± 2.3			
	3/35	7.78 ± 0.12	138.2 ± 0.3	18.6 ± 0.8	0.5 ± 0.1	10.5 ± 1.9			
2	4/15	7.27 ± 0.05	21.3 ± 1.3	25.3 ± 0.9	1.4 ± 0.1	45.8 ± 4.3	7.3	14.0	48.8
	5/25	7.17 ± 0.04	16.1 ± 0.3	22.0 ± 1.6	1.6 ± 0.3	62.5 ± 0.6			
	6/35	7.24 ± 0.02	18.6 ± 0.4	20.7 ± 1.1	1.4 ± 0.1	49.8 ± 1.9			
3	7/15	7.39 ± 0.03	28.8 ± 2.2	23.3 ± 0.9	1.2 ± 0.8	39.3 ± 2.5	7.4	29.8	53.7
	8/25	7.17 ± 0.04	30.6 ± 1.9	23.3 ± 3.9	2.2 ± 0.3	73.6 ± 9.7			
	9/35	7.33 ± 0.02	22.8 ± 0.8	21.1 ± 0.5	1.2 ± 0.02	41.2 ± 0.7			
4	10/35	7.21 ± 0.13	2.8 ± 0.8	2.9 ± 0.5	0.2 ± 0.02	7.5 ± 0.6	7.5	27.6	23.9
5	11/25	7.22 ± 0.18	22.6 ± 0.2	13.1 ± 0.8	0.9 ± 0.02	29.8 ± 0.5	7.6	25.7	25.4
	12/35	7.56 ± 0.02	45.1 ± 0.02	18.3 ± 0.4	0.7 ± 0.02	25.4 ± 0.7			

a ND = nondetecting content, because the collected volume of the ISF was not enough to perform the measurements.

b Approximated time after anesthesia at which the corresponding on-body measurement was performed.

Figure S8a presents the correlation observed for ISF and blood *P*CO_2_, which is the main target analyte herein, as an example. The Pearson correlation coefficients for the relationships between ISF and blood values for pH, HCO_3_^–^ and *P*CO_2_ (*n* = 11 for each parameter) were found to be 0.91, 0.74, and 0.86. A positive correlation between both biofluids (considering a threshold of 0.75) was revealed for pH and *P*CO_2_, while it was less evident for HCO_3_^–^. Analogously, the results from a paired *t* test for pH and *P*CO_2_ parameters pointed out no statistically significant differences (95% confidence interval) between both biofluids (*p* = 0.07 for pH and *p* = 0.97 for *P*CO_2_). Statistical differences were observed for the bicarbonate (*p* = 0.02). Notably, HCO_3_^–^ concentrations were obtained by calculations with both techniques (MNs and i-Stat). For the i-Stat, it has been reported that the provided levels may differ from reality,^33^ which would explain the differences found with the MNs.

Figure S8b presents the Bland-Altman analysis carried out for the ISF-blood *P*CO_2_ (*n* = 11), suggesting a minor bias (the mean difference between the 2 methods) of 0.1 mmHg, with an acceptable precision (SD of the differences) of 10.3 mmHg. The 95% lower and upper limits of agreement were −20.0 and 20.1 mmHg, respectively, englobing all of the samples. A high number of samples showed differences >4.5 or 7.5 mmHg: 45% were ≤4.5 mmHg and 64% ≤ 7.5 mmHg. Only 36% of the measurements were outside the clinically acceptable range (±7.5 mmHg), as recommended by the American Association for Respiratory Care Therapists.^34^

It is important to mention that the statistical analysis was performed comparing ISF *P*CO_2_ at different time points from the anesthesia (ca. 15, 25, and 35 min) with blood *P*CO_2_ obtained after ca. 40 min from the anesthesia, which is not an ideal situation but that was adopted for practicality reasons. In an ideal scenario, blood samples would have been collected 10 min prior to each on-body measurement in ISF to be more comparable between them. Although venous blood sampling from the dorsal pedal vein was performed in our experiments between each interval of the on-body measurements with the patch, insufficient blood volume for analysis with the blood gas analyzer was extracted. This was due to the decrease of blood flow in the rat caused by the anesthesia.^35^

Also, *P*CO_2_ is a dynamic physiological parameter: changes in ISF along the entire assay may be attributed to natural fluctuations in the animal as well as as a consequence of the anesthesia conditions. For example, a decrease in the pH from 7.4 to 7.2 connected to an increment of *P*CO_2_ from ca. 30 to 40 mmHg has been reported in rodents under isoflurane anesthesia for 45 min. Accordingly, it seems logical to compare the values for ISF measurements (except for rat no. 4) at 35 min from the anesthesia with the arterial-venous blood values herein collected, as these are the data closer in time. A paired *t* test (*n* = 4) revealed no statistically significant differences between ISF and blood *P*CO_2_ (*p* = 0.21).

Overall, our results demonstrated not only the possibility of performing accurate intradermal CO_2_ measurements but also a promising correlation between ISF and blood *P*CO_2_. Despite the auspicious outcomes, we believe important to discuss some aspects useful to advance the analytical reliability and technological readiness of the herein developed patch. Regarding the correlation between ISF and blood measurements: (i) To the best of our knowledge, there are no previous reports on correlations between ISF and blood CO_2_. However, a correlation seems to be expected, which is endorsed by existing relationships between both arterial and venous CO_2_ with transcutaneous values.^8^ In any case, a lag time of ca. 10 min should manifest. (ii) Shorter or larger differences have been reported for blood and transcutaneous CO_2_ depending on the measurement position (e.g., earlobe, chest, and arm/forearm).^34^ (iii) Arterial and venous CO_2_ levels correlate between them and hence, mixed blood samples may be an alternative when arterial and/venous blood collection are not feasible, as in our experiments. (iv) The existence of a physiological disagreement between ISF and blood could be possible. Notably, dermal CO_2_ concentrations come from blood diffusion but also CO_2_ production by the tissues, in analogy to transcutaneous CO_2_.^2,5^ Accordingly, at some point, it would be convenient to directly study the relationship between ISF CO_2_ levels and the clinical conditions of interest.

Considering the CO_2_ values provided by the MN patch, several calculations are needed to translate the pH and CO_3_^2–^ measurements into *P*CO_2_, which incorporate summative errors into the outcomes. Importantly, the *K*_a_ values needed to operate eq 1 may change with certain (physio)pathological conditions due to changes in the temperature, ionic strength, and others. The same applies to the solubility product in eq 2, indeed we used that reported for blood and assuming the same value for ISF. On the one hand, calculations will benefit from temperature-based corrections using an MN sensor that could be additionally implemented in the patch. Not only the constants but also the potentiometric slope of the MN sensors could be improved. On the other hand, when performing MN-based measurements in the clinical context in the near future, it would be convenient to look for relationships between pH/CO_3_^2–^ levels with the health conditions rather than CO_2_, to reduce calculations. Alternatively, a HCO_3_^–^ sensor could be used instead of the CO_3_^2–^ one due to the larger levels in blood and thus in ISF.

## Conclusions

We developed an MN-based analytical platform for detecting CO_2_ levels in ISF based on transdermal pH and CO_3_^2–^ measurements. The adequate analytical performances demonstrated at the in vitro level for the pH– and CO_3_^2–^–MN sensors inspired the monitoring of CO_2_ levels in environmentally controlled chambers to validate the needed calculations. Then, ex vivo assays proved the suitability of the CO_2_–MN patch, in terms of resiliency and accuracy. Furthermore, coupling the patch with a portable potentiometer wirelessly connected to a mobile phone for data visualization allowed for in vivo studies in anesthetized rats. Preliminary studies of the correlation between ISF and blood CO_2_ were performed, revealing very interesting prospects. The results herein presented have a great potential for CO_2_ analysis inside organs such as the skin of animals and humans, but also, it could be applied for other tissues and beings (e.g., plants), where CO_2_ monitoring is relevant.

## Supporting Information Available

Experimental details; procedures for in vivo tests; pictures of the system; SEM images; reproducibility; selectivity; and drift (PDF)
