# Supplementary material for: Demonstrating the Analytical Potential of a Wearable Microneedle-Based Device for Intradermal CO2 Detection
Source: ACS Sens. 2024 Jan 4;9(1):361–70. doi: 10.1021/acssensors.3c02086 (PMC10825866; doi:10.1021/acssensors.3c02086)
Supplement: Supplementary file 1 — se3c02086_si_001.pdf [file se3c02086_si_001.pdf]

Supporting Information for:

Demonstrating the Analytical Potential of a Wearable Microneedle-based Device for Intradermal CO_2 Detection

Águeda Molinero-Fernandez[1,2], Qianyu Wang[1], Xing Xuan[1,2], Åsa Konradsson-Geuken[3], Gastón A. Crespo[1,2] and María Cuartero[1,2,*]

[1]Department of Chemistry, KTH Royal Institute of Technology, Teknikringen 30, SE-100 44, Stockholm, Sweden.

[2]UCAM-SENS, Universidad Católica San Antonio de Murcia, UCAM HiTech, Avda. Andres Hernandez Ros 1, 30107, Murcia, Spain.

[3] Section of Neuropharmacology and Addiction Research, Department of Pharmaceutical Biosciences, Uppsala University, Uppsala, Sweden.

**Corresponding authors: Maria Cuartero (mariacuartero@ucam.edu); Gaston A. Crespo (gacp@kth.se)*

Table of Contents

Experimental Section S2
Reagents, materials and instrumentation. S2
Composition of the membrane. S2
Potentiometric measurements. S2
Selectivity studies. S2
Dynamic CO_2 measurements. S3
Ex vivo assays. S3
In vivo assays. S3
Tables S4
Table S1 ..... S4
Table S2 ..... S4
Table S3 ..... S4
Table S4 ..... S5
Table S5 ..... S5
Table S6 ..... S6
Table S7 ..... S6
Figures S7
Figure S1 ..... S7
Figure S2 ..... S7
Figure S3 ..... S7
Figure S4 ..... S8
Figure S5 ..... S8
Figure S6 ..... S8
Figure S7 ..... S9
Figure S8 ..... S9
References S10

1. Experimental Section

Reagents, materials and instrumentation. Analytical grade chloride salts of potassium, sodium, calcium, magnesium and lithium as well as sodium carbonate, sodium phosphate, sodium citrate, bovine serum albumin (BSA), urea and glucose were also purchased from Sigma-Aldrich. CO_2 cylinder (20 L) was purchase from Salanders åkeri.

The synthesis of lipophilic functionalized multiwalled-carbon nanotubes (f-MWCNTs) was performed as reported elsewhere.[1] For this purpose, thionyl chloride (SOCl2 ≥ 97% purity), octadecylamine (ODA, > 99%), dimethylformamide (DMF, > 99%) and MWCNTs (from Sigma Aldrich) were used.

The artificial interstitial fluid employed for in-vitro assays was prepared with 3.5 mM KCl, 1.5 mM $CaCl_2$, 0.7 mM $MgCl_2$, 140 mM NaCl, 26 mM $NaHCO_3$, 1.7 mM Na_2HPO_4, 6 mM glucose and 7 mM urea. The target ions were removed from the initial recipe.

Electromotive force (EMF) was measured with a high input impedance (1015) EMF16 multichannel data acquisition device (Lawson laboratories, Inc.) against a double junction Ag/AgCl/sat. KCl/1M LiOAc reference electrode (Metrohm Nordic, Sweden). Scanning Electron Microscopy (SEM) images were taken with a Zeiss Merlin VP Compact SEM (Zeiss, Inc.). Micro-pH meter (LL, biotrode, Metrohm, Nordic Sweden), and ultra-micro pH meter (Orion, Ultra-Micro Combination pH Electrode, Thermo Scientific) were employed for the CO_2 chamber experiments, and subcutaneous and ISF pH measurements, respectively. Portable blood gas analyzer ABG i-STAT 1 (Abbott), and cartridges CG4+ (Abbott) were employed for blood analysis in in vivo experiments.

Composition of the membranes. The cocktail for the pH-selective membrane was prepared by dissolving 1.1 mg of hydrogen ionophore I, 0.3 mg of KTClPB, 65.8 mg of o-NPOE and 32.9 mg of PU in 1 mL of THF.[2] For the carbonate membrane, 4.1 mg of carbonate ionophore VII, 1 mg of TDMACl, 50 μL of EHA and 30 mg of PVC were dissolved in 1 mL of THF.[3] The reference membrane cocktail was prepared by dissolving 78 mg of PVB and 50 mg NaCl in 1 ml of methanol.[4]

Potentiometric measurements. Calibration experiments were carried out at room temperature (22 ± 1 °C) under constant stirring of 300 rpm (stirrer IKA COLOR SQUID S000, IKA, Germany). The MN-based electrodes (WEs and RE) were connected to the potentiometer by a cable based on electrical clamps and BNC outputs. In the case of rat-based experiments, a hand-made potentiometric board was used.

Selectivity studies. To obtain the individual calibration graphs for the main and interfering ions, the activity coefficients in the measured solutions were calculated using a two-parameter Debye-Hückel approximation from the experimental concentrations.[5] Each logarithmic activity was plotted against the corresponding steady-state potential, and the curves were fitted to the

Nernst equation.[6] Selectivity was evaluated using the separate solution method (SSM) according to Bakker et al.[7] Individual calibration graphs were experimentally accomplished at increasing activities of the primary and the interfering cations. The logarithmic selectivity coefficients were calculated by extrapolating the response to $a_{I,J} = 1\ M$, using the portion of the calibration curve close to Nernstian response.

Dynamic CO_2 measurements. A hand-made plastic chamber (59,0 x 39,5 x 41,2 cm) was employed for dynamic CO_2 measurements of samples with unknown concentrations. The CO_2 cylinder was connected to the chamber by a gas tubing. Inside the chamber the solution (containing $NaHCO_3$ or AISF) with all the sensors was placed, and 100% CO_2 gas flow was bubbling into the chamber. All the experiments were conducted at 25ºC into a room with temperature control.

***Ex vivo* measurements**. To test the mechanical resilience of the CO_2-MN patch, an experiment consisting of inserting the MNs into a piece of rat skin and removing it was performed after an initial calibration graph. The, more calibrations were accomplished after each insertion, being compared with the initial one to evaluate the resiliency towards on-body measurements. Here, the insertion force was manually applied during each cycle.

Next, the CO_2 level was monitored inside pieces of rat skin previously conditioned with different CO_2 concentrations. The conditioned skin was fixed in a 3D-printed holder and the MN patch was inserted into the skin to register the potential. The potential was converted into concentration by means of a previous calibration graph. Finally, ISF (i.e., liquid inside the piece of skin) was collected using a home-made extraction system composed of a hollow MN hub connected to a syringe (**Figure S7**) and then a syringe pump. The ISF sample was analyzed by the ultra-micro-pH meter, the CO_3^{2-}-MN and/or the i-STAT 1 system.

***In vivo* measurements**. Five male Wistar rats (Envigo, the Netherlands) weighing approximately 180 grams on arrival (~six weeks of age) from a set of twelve rats were used for the *in vivo* MN-based measurements. Animals were maintained in groups of four under standard laboratory conditions (room temp of ~21 ℃ and relative humidity of 55-65 %) with a 12 h reversed light/dark cycle (lights on at seven PM) and *ad libitum* access to food and water. During the experiment, rats were anaesthetized with isoflurane. After the MN-based experiment, each animal got a lethal dose of isoflurane followed by decapitation. A mixture of arterial-venous blood samples was immediately collected and analyzed after the euthanasia, minimizing the contact with the air. The i-Stat blood gas analyzer was used. Also, whole blood samples from rats #1 and #3 were collected and stored in a commercial sample collector that contains lithium heparin to avoid coagulation, being measured after sample equilibrium with atmospheric CO_2 (approx. 3 hours). For *in-vivo* CO_2 calculation 6.1, and 10.22 were employed as pka_1, and pka_2, and 0.03 mmol/L as S.[8,9] Bicarbonate concentration was calculated from pH and carbonate concentrations measured experimentally with the MN patch. A value of 10.22 was used as the pKa_2 in the following equation *(Equation S1)*.[9,10]

$$\text{Log}\,[HCO_3^-] = pKa_2 + \text{Log}\,[CO_3^{2-}] - pH \qquad \text{(Equation S1)}$$

2. Tables

Table S1. Comparison of concentrations expected in arterial blood, venous blood, and ISF.[9,11,12]

Analyte	Concentrations (mM)		
	Arterial	Venous	ISF
Bicarbonate (HCO_3^-)	25.40	27.66	31
Dissolved CO_2	1.05	1.23	1.8 (50 mmHg)[a]
Carbonate (CO_3^{2-})[b]	0.043	0.044	0.049
pH	7.5	7.4	7.4

[a]Value expressed as PCO_2 in mmHg.
[b]Calculated from the CO_2 concentration.

Table S2. Variation of the calibration parameters observed in the reproducibility and repeatability evaluation of the MN-based patch.

Analyte	Electrode	Repeatability		Reproducibility	
		Slope (mV)	Intercept (mV)	Slope (mV)	Intercept (mV)
pH	RE_{com}	–53.9 ± 1.0	521.1 ± 5.0	–53.3 ± 1.0	591.1 ± 5.4
	RE_{MN}	–55.5 ± 0.3	697.2 ± 5.0	–50.7 ± 0.8	501.5 ± 5.7
CO_3^{2-}	RE_{com}	–25.8 ± 0.3	95.3 ± 0.6	–26.9 ± 0.9	35.9 ± 9.0
	RE_{MN}	–28.4 ± 0.9	25.17 ± 4.9	–23.5 ± 1.9	76.9 ± 20.2

Table S3. Analytical parameters obtained for pH and CO_3^{2-}.

Analyte	RE	Sensitivity (mV dec^{-1})	Intercept (mV)	LOD	LRR (M)	Response time (s)
pH	$RE_{comm.}$	–53.9 ± 1.0	521.1 ± 5.0	–	8.5 – 5.0 (pH units)	2.5 – 1.5
	RE-MN	–55.5 ± 0.3	697.2 ± 5.0	–	8.5 – 5.0 (pH units)	3.0 – 1.5
CO_3^{2-}	$RE_{comm.}$	–25.8 ± 0.3	95.3 ± 0.6	$10^{-5.9}$	$10^{-5.4}$ – $10^{-2.5}$	3.5 – 2.5
	RE-MN	–28.4 ± 0.9	25.2 ± 4.9	$10^{-5.8}$	$10^{-5.5}$ – $10^{-2.6}$	3.5 – 2.5

Table S4. Electrolyte concentrations typically found in human interstitial fluid.[13]

Analyte	Concentration (mM)
pH	ca. 7.4[a]
Na^+	135.7
K^+	3.4
Ca^{2+}	1.1
Cl^-	115
HCO_3^-	31
CO_3^{2-}	0.049
$H_2PO_4^-$	1.3
SO_4^{2-}	0.6

[a]pH units

Table S5. Logarithmic selectivity coefficients for main interferences calculated from the MN measurements together with those reported in the literature for analogous MNs and electrodes. I=main ion. J=interfering ion.

Ions (I,J)	This paper	pH-MN[2]	pH-MN[14]	pH ISE[15]	Reported concentrations[16,17]	Needed Value
H^+, K^+	–5.2 ± 0.06	–4.9 ± 0.07	–6.7 ± 0.08	–9.3 ± 0.8	3.5 mM	–1.8
H^+, Na^+	–4.5 ± 0.05	–4.7 ± 0.09	–6.0 ± 0.09	–10.2 ± 0.1	152.3 mM	–3.9
H^+, Ca^{2+}	–4.0 ± 0.07	–4.1 ± 0.06	–5.0 ± 0.09	–10.4 ± 0.1	1.6 mM	–2.9
**Ions (I,J)**	**This paper**	**CO_3^{2-}-MN**		**CO_3^{2-} ISE**	**Reported concentrations[16,17]**	**Needed Value**
CO_3^{2-}, Cl^-	–5.1 ± 0.09	–4.2		-6.8	109.0 mM	–2.9

Table S6. *In vitro* monitoring of CO_2 in artificial $NaHCO_3$ and AISF samples. Diff=% of difference between the results provided by the two techniques.

Number	Sample	CO_2-MN (mM)	Severinghaus Probe (mM)	Diff. (%)
#1	$NaHCO_3$	0.4[a]	0.4	0
#2	$NaHCO_3$	0.6[a]	0.7	14
#3	$NaHCO_3$	3.1	2.9	7
#4	$NaHCO_3$	3.8	3.6	6
#5	$NaHCO_3$	8.8	8.6	2
#6	$NaHCO_3$	10.8	12.6	14
#7	$NaHCO_3$	15.5	13.9	12
#8	$NaHCO_3$	24.3	35.3	31
#9	AISF	1.6[a]	1.8	10
#10	AISF	1.9[a]	2.2	14
#11	AISF	9.3	8.1	15
#12	AISF	13.4	12.7	5
#13	AISF	17.0	19.8	14
#14	AISF	27.7	28.9	4
#15	AISF	32.7	39.9	18

[a]The measurement was accomplished before the CO_2 flowing in the chamber.

Table S7. pH and blood gas values after equilibration with the atmosphere.

Rat number (#)	Blood sample	pH	PCO_2, mmHg
**#1**	Before	7.70	18.8
	After	8.10	7.9
**#3**	Before	7.35	53.7
	After	7.6	28.7

3. Figures

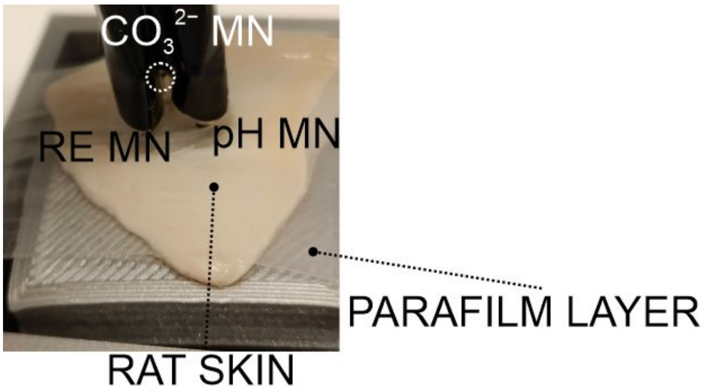

Figure S1. Picture of the setup for *ex vivo* experiments.

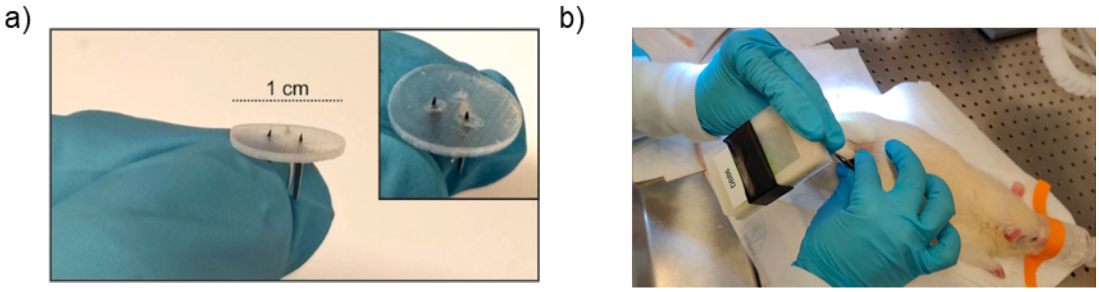

Figure S2 Pictures of: (a) the CO_2-MN patch, and (b) on-body measurements with the sensing device.

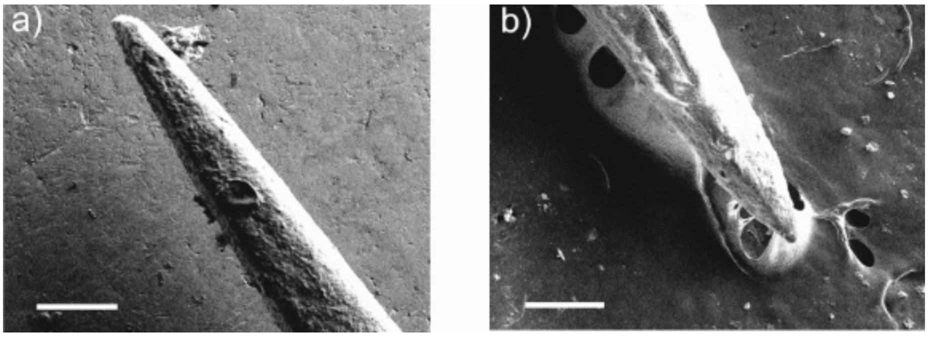

Figure S3. SEM images of the: (a) CO_3^{2-}-MN and (b) RE-MN. Scale bars: 100 μm.

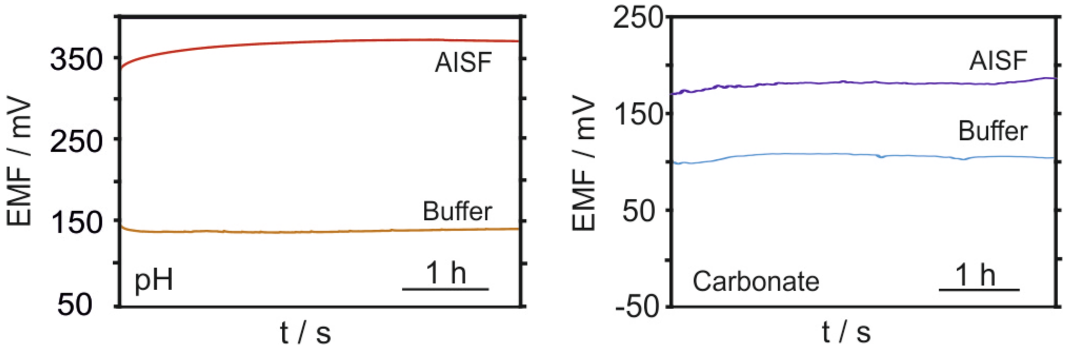

Figure S4. Medium-term stability study of the pH- and CO_3^{2-}-MN sensors performed in buffer and AISF media.

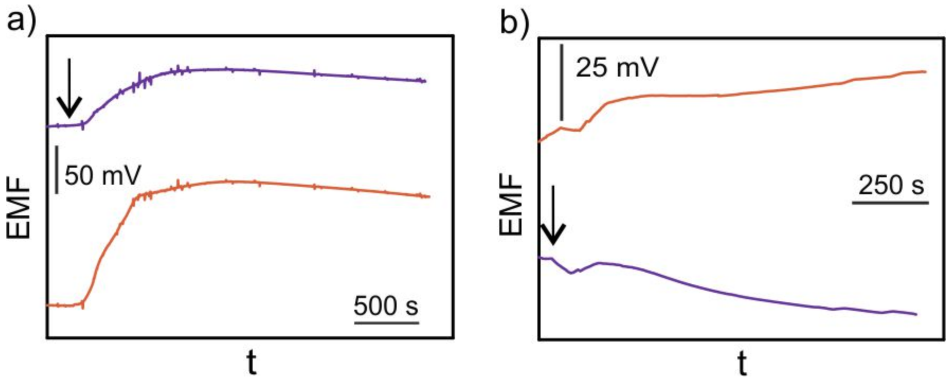

Figure S5. Dynamic pH (red), and carbonate (purple) MN responses during *in vitro* monitoring of CO_2 in the chamber in artificial $NaHCO_3$ (a) and AISF samples (b). The arrows indicate the moment that the CO_2 gas flow initiated.

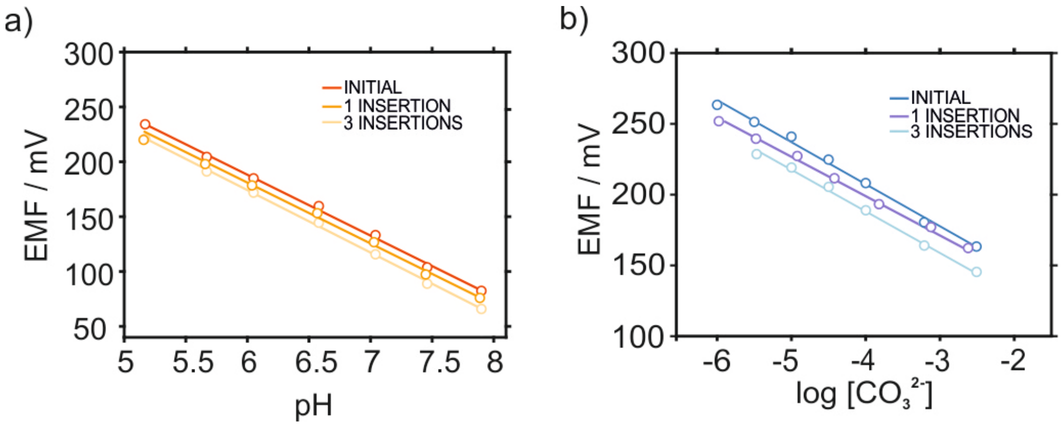

Figure S6. Calibration graphs observed for pH and CO_3^{2-} before and after several insertions into rat skin.

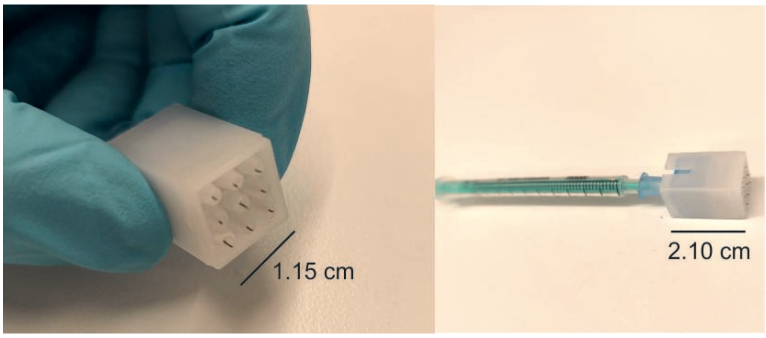

Figure S7. Home-made device based on a hollow MN-hub connected to a syringe for ISF collection.

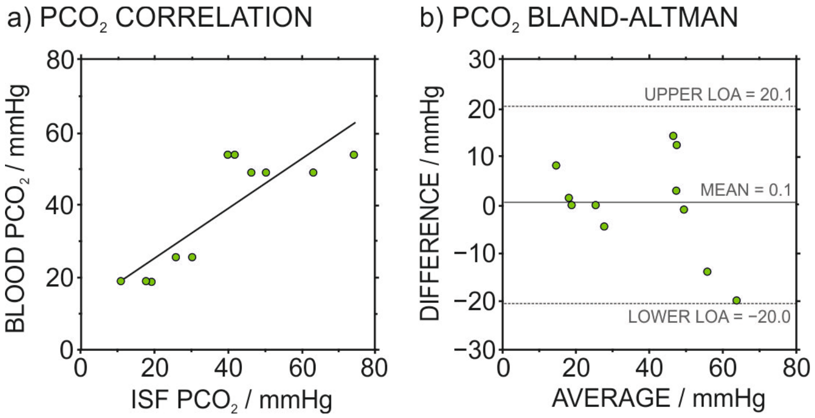

Figure S8. ISF and blood PCO_2 relationship. (a) Correlation. (b) Bland–Altman plot. The mean difference (“bias”) and ±1 standard deviations are represented with solid and dash lines respectively.

4. References

(1) Yuan, D.; Anthis, A. H. C.; Ghahraman Afshar, M.; Pankratova, N.; Cuartero, M.; Crespo, G. A.; Bakker, E. All-Solid-State Potentiometric Sensors with a Multiwalled Carbon Nanotube Inner Transducing Layer for Anion Detection in Environmental Samples. *Anal Chem* **2015**, *87* (17), 8640–8645.

(2) Molinero-Fernández, Á.; Casanova, A.; Wang, Q.; Cuartero, M.; Crespo, G. A. In Vivo Transdermal Multi-Ion Monitoring with a Potentiometric Microneedle-Based Sensor Patch. *ACS Sens* **2023**, *8* (1), 158–166.

(3) Cuartero, M.; Crespo, G.; Cherubini, T.; Pankratova, N.; Confalonieri, F.; Massa, F.; Tercier-Waeber, M.-L.; Abdou, M.; Schäfer, J.; Bakker, E. In Situ Detection of Macronutrients and Chloride in Seawater by Submersible Electrochemical Sensors. *Anal Chem* **2018**, *90* (7), 4702–4710.

(4) Guinovart, T.; Crespo, G. A.; Rius, F. X.; Andrade, F. J. A Reference Electrode Based on Polyvinyl Butyral (PVB) Polymer for Decentralized Chemical Measurements. *Anal Chim Acta* **2014**, *821*, 72–80.

(5) Meier, P. C. Two-Parameter Debye-Hückel Approximation for the Evaluation of Mean Activity Coefficients of 109 Electrolytes. *Anal Chim Acta* **1982**, *136*, 363–368.

(6) Bakker, E.; Pretsch, E. Modern Potentiometry. *Angewandte Chemie International Edition* **2007**, *46* (30), 5660–5668.

(7) Bakker, E.; Pretsch, E.; Bühlmann, P. Selectivity of Potentiometric Ion Sensors. *Anal Chem* **2000**, *72* (6), 1127–1133.

(8) Rivkees, S. A.; Fine, B. P. The Reliability of Calculated Bicarbonate in Clinical Practice. *Clin Pediatr (Phila)* **1988**, *27* (5), 240–242.

(9) KRUSE, J. A. Calculation of Plasma Bicarbonate Concentration *versus* Measurement of Serum CO_2 Content. PK′ Revisited. *Clinical Intensive Care* **1995**, *6* (1), 15–20.

(10) Wietasch, K.; Kraig, R. P. Carbonic Acid Buffer Species Measured in Real Time with an Intracellular Microelectrode Array. *Am. J. Physiol. - Regul. Integr. Comp. Physiol.* **1991**, 261, R760-R765.

(11) Murray, C. D.; Hastings, A. B. The maintenance of carbonic acid equilibrium in the body, with especial reference to the influence of respiration and kidney function on co2, h+, hco3', and co3" concentrations in plasma. *Journal of Biological Chemistry* **1925**, *65*, 265–278.

(12) Magder, S.; Magder, A.; Samoukovic, G. Intracellular PH Regulation and the Acid Delusion. *Can J Physiol Pharmacol* **2021**, *99* (6), 561–576.

(13) Fogh-Andersen, N.; Altura, B. M.; Altura, B. T.; Siggaard-Andersen, O. Composition of Interstitial Fluid. *Clin Chem* **1995**, *41* (10), 1522–1525.

(14) García-Guzmán, J. J.; Pérez-Ràfols, C.; Cuartero, M.; Crespo, G. A. Toward *In Vivo* Transdermal PH Sensing with a Validated Microneedle Membrane Electrode. *ACS Sens* **2021**, *6* (3), 1129–1137.

(15) Novell, M.; Parrilla, M.; Crespo, G. A.; Rius, F. X.; Andrade, F. J. Paper-Based Ion-Selective Potentiometric Sensors. *Anal Chem* **2012**, *84* (11), 4695–4702.

(16) Gilanyi, M.; Ikrenyi, C.; Fekete, J.; Ikrenyi, K.; Kovach, A. G. Ion Concentrations in Subcutaneous Interstitial Fluid: Measured versus Expected Values. *American Journal of Physiology-Renal Physiology* **1988**, *255* (3), F513–F519.

(17) Bretag, A. H. Synthetic Interstial Fluid for Isolated Mammalian Tissue. *Life Sci* **1969**, *8* (5), 319–329.
